# HIV-1–infected T cell clones are shared across cerebrospinal fluid and blood during ART

**DOI:** 10.1172/jci.insight.176208

**Published:** 2024-04-08

**Authors:** Meng Wang, Jennifer Yoon, Hailey Reisert, Bibhuprasad Das, Benjamin Orlinick, Jennifer Chiarella, Elias K. Halvas, John Mellors, Alina P.S. Pang, Lydia Aoun Barakat, Margaret Fikrig, Joshua Cyktor, Yuval Kluger, Serena Spudich, Michael J. Corley, Shelli F. Farhadian

**Affiliations:** 1 Program in Computational Biology and Bioinformatics, Yale University, New Haven, Connecticut, USA.; 2 Section of Infectious Diseases, and; 3 Department of Neurology, Yale School of Medicine, New Haven, Connecticut, USA.; 4 Department of Medicine, University of Pittsburgh, Pittsburgh, Pennsylvania, USA.; 5 Department of Medicine, Division of Infectious Diseases, Weill Cornell Medicine, New York City, New York, USA.; 6 Department of Pathology, Yale School of Medicine, New Haven, Connecticut, USA.; 7 Department of Epidemiology of Microbial Diseases, Yale School of Public Health, New Haven, Connecticut, USA.

**Keywords:** AIDS/HIV, Immunology, T cell receptor

## Abstract

The central nervous system HIV reservoir is incompletely understood and is a major barrier to HIV cure. We profiled people with HIV (PWH) and uninfected controls through single-cell transcriptomic and T cell receptor (TCR) sequencing to understand the dynamics of HIV persistence in the CNS. In PWH on ART, we found that most participants had single cells containing HIV-1 RNA, which was found predominantly in CD4 central memory T cells, in both cerebrospinal fluid (CSF) and blood. HIV-1 RNA–containing cells were found more frequently in CSF than blood, indicating a higher burden of reservoir cells in the CNS than blood for some PWH. Most CD4 T cell clones containing infected cells were compartment specific, while some (22%) — including rare clones with members of the clone containing detectable HIV RNA in both blood and CSF — were found in both CSF and blood. These results suggest that infected T cells trafficked between tissue compartments and that maintenance and expansion of infected T cell clones contributed to the CNS reservoir in PWH on ART.

## Abstract 2

Single-cell TCR profiling of CSF and blood cells of people with HIV reveals shared infected T cell clones across tissue even after ART.

## Introduction

HIV establishes cellular reservoirs during acute infection, with these reservoirs persisting even after years of antiretroviral therapy (ART) (1–3). The central nervous system (CNS) is seeded by HIV within days of infection, and autopsy studies of people who died while on ART suggest that infected cells persist in the CNS even after years of ART (4–8). HIV persistence in the CNS is, therefore, a major barrier to HIV cure. HIV persistence may also have clinical consequences for people with HIV (PWH), since, despite undergoing suppressive ART, some PWH still manifest CNS abnormalities, including cognitive impairment, stroke, and mental health disorders (9–13). These HIV-associated CNS complications may be augmented by persistent viral replication within the CNS (14–18). There is, therefore, an urgent need to understand the dynamics of HIV persistence in the CNS in PWH and a specific need to understand these processes in PWH on ART.

Cerebrospinal fluid (CSF) bathes the brain and serves as a critical immune surveillance site of the CNS that is directly accessible in living people (19). CSF studies of PWH have provided invaluable insights into HIV dynamics during acute and chronic infection and suggest ongoing and abnormal CNS immune activation in PWH on suppressive ART (4, 20). Prior studies of the CSF in PWH identified abundant HIV transcripts in CSF during acute infection (21), with persistent traces of HIV DNA and RNA found in CSF cells even among PWH on suppressive ART (16, 22). Studies focused on CSF-derived viral sequences suggest that CSF contains both macrophage and T cell tropic viruses; that some CSF CD4 T cells express HIV p24 antigen, even during ART; and that host cell markers on HIV virions derived from CSF are of T cell origin (23–27). These findings underscore the potential role of CSF T cells as key sites of HIV persistence. However, it remains unclear which specific CSF T cell subsets harbor HIV during ART. Furthermore, the dynamics of these latently infected CSF T cells — whether they become compartmentalized and clonally expand within the CNS or maintain cellular trafficking with peripheral blood — remains incompletely understood.

The advent of single-cell RNA-Seq (scRNA-Seq) paired with T cell receptor (TCR) sequencing offers a powerful tool to examine the transcriptional states and clonality of T cells at a single-cell resolution and across compartments (28–30). In this study, we utilized scRNA-Seq alongside TCR sequencing of paired blood and CSF to track individual T cell clones between CSF and blood and to investigate the presence of transcriptionally active HIV-1 RNA–producing T cells in CSF and blood of PWH on suppressive ART. By tracing both infected and uninfected clones between CSF and blood, we sought evidence for compartmentalization of infected T cells. Such insights are pivotal in deepening our understanding of the HIV reservoir in the CNS and in developing effective strategies for HIV cure.

## Results

### Characteristics of study participants.

We enrolled 8 PWH, labeled P1–P8. P1 was an ART-naive individual diagnosed with AIDS (CD4 T cell count 47 cells/μL) and concurrent neurosyphilis at his initial study visit. This individual was closely monitored and sampled multiple times: before starting ART and then again at 3, 7, and 9 months after commencing ART. We collected samples at a single time point from the remaining 7 PWH (P2–P8) who were neurologically asymptomatic and had been on stable suppressive ART for an average duration of 16 years. Paired CSF and blood were also analyzed from 6 HIV-uninfected individuals who were demographically similar, labeled C1–C6 (Supplemental Tables 1 and 2; supplemental material available online with this article; https://doi.org/10.1172/jci.insight.176208DS1). All participants consented to undergo lumbar puncture and provide blood samples for research studies.

### Distinct immune cell type composition between blood and CSF.

**Figure 1:**
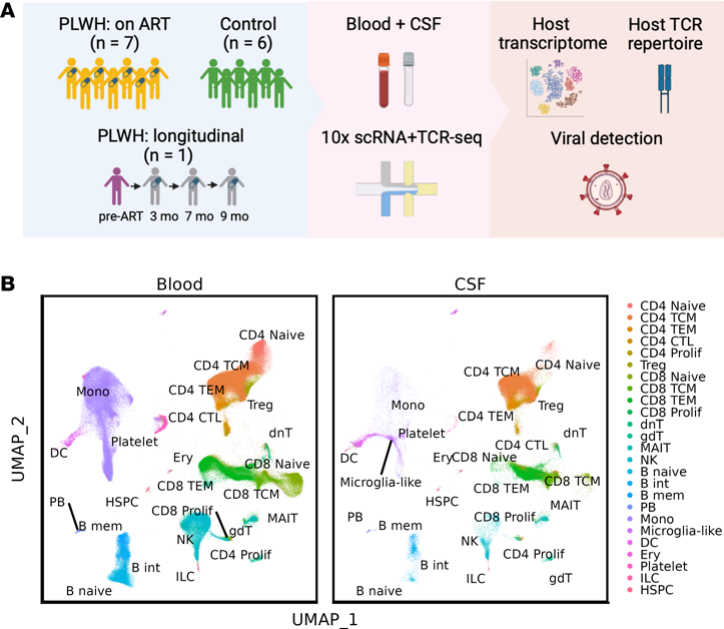
Experiment pipeline and cell type annotations. (**A**) The 10× paired single-cell RNA-Seq and TCR-Seq were performed on the blood and CSF samples collected from 8 PWHs (7 on ART, 1 off ART at the first visit) and 6 healthy controls. (**B**) UMAP plots from gene expression data of all samples (combined), PBMC cells (blood), and CSF cells. Cell types were annotated using Azimuth based on Human PBMC reference data. TE, effector memory T cells; CTL,cytotoxic T cells; Prolif, proliferating T cells; dnT, double-negative T cells; gdT, γ-δ T cells; MAIT, mucosal-associated invariant T cells, int, intermediate; B mem, memory B cells; PB, plasmablasts; Mono, monocytes; Microglia-like, CD204^+^ microglia-like cells; Ery, erythroid cells; ILC, innate lymphoid cells; HSPC, hematopoietic stem and progenitor cell.

To characterize immune cell subsets within the CNF and blood, we measured the gene expression and TCRs of the peripheral blood and CSF samples using 5′ V(D)J 10× Genomics paired scRNA-Seq and TCR-Seq (Figure 1A and Supplemental Tables 3 and 4). In total, we examined the single-cell transcriptomes of 129,544 CSF cells and 262,818 PBMCs from PWH and uninfected controls. We annotated the immune cell types using the reference-based mapping pipeline Azimuth (31), refined for unique CSF myeloid populations based on our prior studies of CSF single-cell transcriptomes (Figure 1B and Supplemental Figure 1), and compared cell type frequencies between tissue compartments (Supplemental Figure 2). We observed a significant difference in cell type frequency composition between blood and CSF (χ^2^ test, *P* < 0.01). Consistent with prior research (22, 29), CSF is mainly composed of T cells (84.6%), a much higher percentage compared with blood (52.9%; Wilcoxon ranked-sum test, *P* < 0.001). On the other hand, blood exhibited a larger presence of monocytes, NK cells, and B cells compared with CSF (19.3% versus 2.2%, 10.4% versus 2.6%, 3.7% versus 0.6%, respectively; Wilcoxon ranked-sum test, *P* < 0.001). However, when comparing the PWH on suppressive ART (P2–P8) with uninfected controls, no significant differences were found in cell type frequency for either blood or CSF (χ^2^ tests, *P* > 0.5).

### A low frequency of HIV-1 RNA^+^ transcripts were identified in CD4 T cells in both blood and CSF.

To identify the presence of transcriptionally active HIV-1 RNA^+^ cells in blood and CSF, we aligned the single-cell transcriptome sequencing reads against the HIV-1 genome. For participants P2–P8, we used the HIV-1 HXB2 reference genome (GenBank: K03455.1). For participant P1, who had active viral replication at the time of study entry, we first sequenced his autologous HIV-1 proviral sequences found in his pre-ART plasma. We then aligned the single-cell CSF reads against his autologous HIV-1 viral RNA–generated sequence.

**Figure 2:**
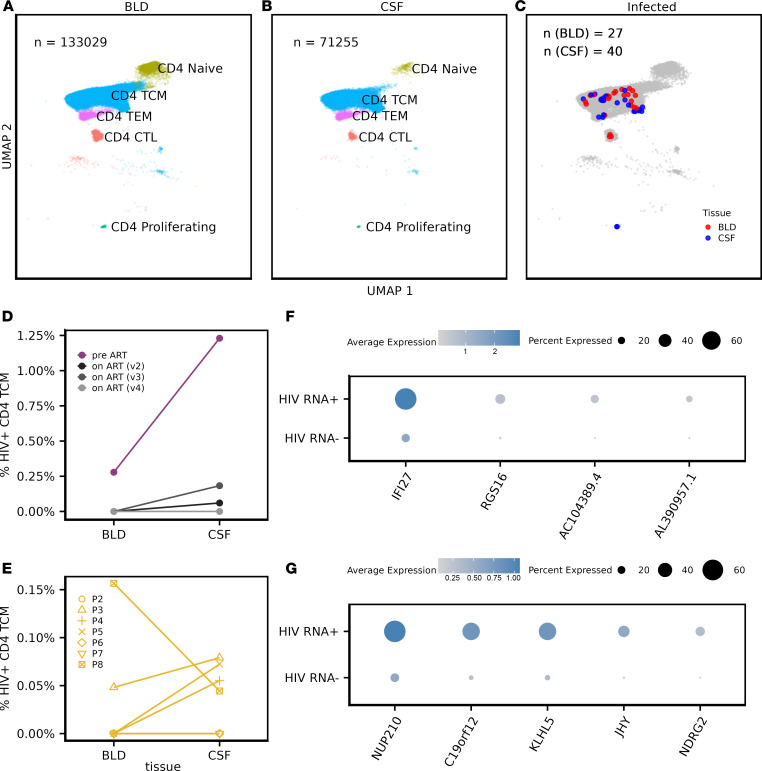
Identifying HIV RNA^+^ CD4 T cells. (**A**–**C**) UMAP plot of CD4 T cells in blood (**A**) and CSF (**B**) as well as T cells with HIV RNA transcripts detected (red, blood; blue, CSF) (**C**). (**D** and **E**) The frequency of HIV-1 RNA^+^ CD4 TCM in longitudinally tracked participant P1 at visit 2–4 (3, 7, 9 months) (**D**) and participants on suppressive ART (**E**). (**F** and **G**) Significantly differentially expressed genes in HIV RNA^+^ compared with HIV RNA^–^ CD4 central memory CSF T cells in P1 (**F**) and P2–P8 (**G**). Wilcoxon ranked-sum test was used with Bonferroni correction to filter for the genes with fold change > 0.25, FDR < 0.05.

In total, across all cells from PWH in our study, we detected HIV-1 transcripts in 85 cells, which represents 0.03% of all single cells we studied, spanning blood and CSF. These cells were found in 8 of 11 CSF samples and 6 of 11 blood samples from PWH (Supplemental Table 5 and Supplemental Figure 4). Notably, among infected CD4 T cells in PWH (Figure 2, A–C, and Supplemental Figure 3), a majority (83.6%) were identified as CD4 central memory T cells (TCM). Participant P1, who was not yet on ART at study initiation, had a higher level of HIV^+^ CD4 TCM cells in the CSF than in the blood before starting ART as well as 3 months and 7 months after ART (Figure 2D). For P1, no HIV^+^ CD4 TCM cells were detected at 9 months after ART initiation in either blood or CSF. In the group of PWH on suppressive ART (P2–P8), 5 of 7 had HIV-1 RNA^+^ CD4 TCM cells detected in both CSF and blood. In these participants, 3 of 5 showed a higher frequency of HIV-1^+^ CD4 TCM cells in CSF than blood (Figure 2E). No HIV-1 transcripts were found in any uninfected control samples.

To better understand the biological signatures of CD4 TCM cells that produce HIV-1 RNA^+^ in the CSF, we conducted a differential expression analysis between the CSF CD4 TCM cells with and without HIV transcripts. We found few genes that were significantly differentially expressed in HIV-1 RNA–producing CD4 TCM cells compared with cells without HIV-1 RNA^+^. In P1 (viremic individual), 4 host genes were significantly upregulated in CSF CD4 TCM cells containing HIV transcripts (fold change > 0.25; FDR < 0.05) (Figure 2F). This included a gene involved in interferon responses (IFI27) and in the p53 pathway (RGS16) (adjusted *P* < 0.05). In P2–P8 (PWH on suppressive ART), 5 host genes were significantly upregulated in HIV-1 RNA–producing CSF CD4 TCM (Figure 2G), including the nucleoporin gene NUP210, previously associated with viral infection in vitro (32).

### Clonal overlap between blood and CSF T cells in PWH.

**Figure 3:**
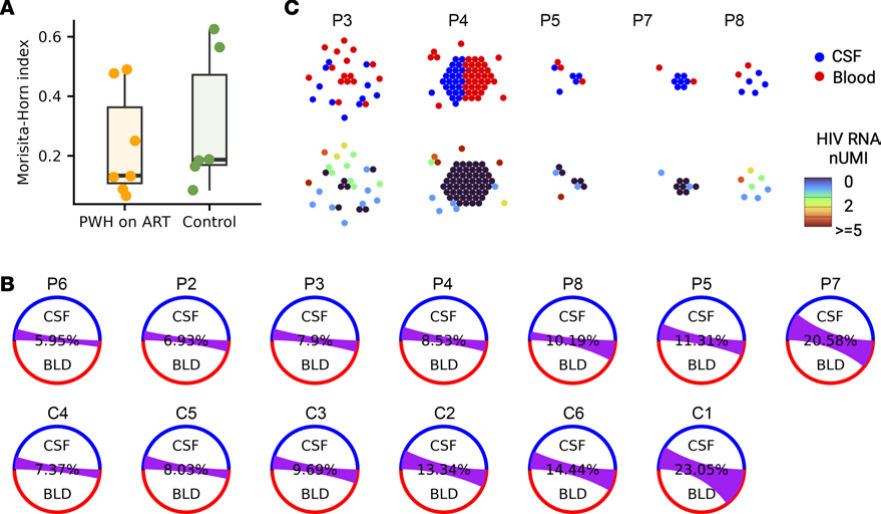
TCR repertoire comparison between PWH on ART and healthy controls. (**A**) Box plot of MH overlap index between blood and CSF TCR clones for healthy controls C1–C7 and PWH on ART P2–P8. Higher MH index indicates greater clonal sharing between compartments. Welch 2 sample *t* test was performed to test for the difference in the MH index with *P* = 0.69. (**B**) Circos diagram showing the clonal overlap between blood and CSF T cells for PWH and uninfected controls, ordered by the level of overlap. The purple chords correspond to cells in shared clones between blood and CSF. (**C**) Honeycomb diagram showing the clonal relationship between clones containing T cells with HIV RNA in PWH on ART. Each cell is represented as a dot. Dots touching each other belong to the same clone and are plotted as a hexagon. The colors represent the compartment and the UMI counts of HIV transcripts for each cell, respectively, for the top and bottom panel.

To evaluate whether there is overlap of T cell clones between the CSF and blood, we used the Morisita-Horn (MH) overlap index to compare TCR repertoires of both compartments. A higher MH index (close to 1) implies a greater clonal overlap between the CSF and blood, while a lower index (close to 0) suggests an increased presence of compartment-unique clones. The degree of clonal overlap varied widely among individuals, with some showing minimal overlap between blood and CSF TCRs and others showing moderate overlap (Figure 3, A and B). We observed a slightly lower clonal overlap in PWH on ART (mean MH of 0.23) compared with HIV-uninfected controls (mean MH of 0.30), meaning that there were marginally more compartment-specific clones in the CSF of PWH. However, this difference was not statistically significant (Figure 3A; Welch 2-sample 2-tailed *t* test, *P* = 0.69).

Next, leveraging single-cell TCR data for paired blood and CSF samples, we compared the infected T cell clones between the 2 compartments. Specifically, we assessed the CD4 TCR clones from cells identified as containing HIV-1 RNA in the 5 PWH on ART who had cells identified as containing HIV-1 RNA in both CSF and blood (Figure 3C). We found 36 CD4 T cell clones that contained at least 1 infected T cell. Most (28 of 36; 78%) of these CD4 T cell clones containing infected cells were compartment specific, while some (8 of 36; 22%) T cell clones containing infected cells were found in both CSF and blood. Most infected CD4 T cells belonged to singletons (unique TCR clones), but 10 of 36 (28%) belonged to CD4 TCR clones with evidence of clonal expansion. Among the T cell clones containing infected cells with members of the clone located across tissue compartments, we observed two CD4 T cell clones (1 in P3 and 1 in P4) with separate single cells in blood and CSF containing detectable HIV RNA. These results suggest that infected CD4 T cells traffic between tissue compartments and provide potential evidence that clonal expansion of infected T cells may occur within the CSF despite ART.

To better understand potential antigenic drivers of T cell clonal expansion in the CSF, we examined the predicted antigen specificity of TCRs in the CSF. We queried known databases, including VDJdb (https://vdjdb.cdr3.net/), McPAS (http://friedmanlab.weizmann.ac.il/McPAS-TCR/), and IEDB (https://www.iedb.org/), for exact TCRβ CDR3 sequence matches (Supplemental Figure 5). On average, 4.1% and 4.2% of the blood and CSF TRB repertoires were annotated. Among these annotated TRB sequences, the most prevalent were those related to *C. albicans* (0.68% of all annotations), cytomegalovirus (CMV) (0.68%), and varicella zoster virus (VZV) meningoencephalitis (0.61%). No significant differences in TCR epitope species composition were observed in the blood versus the CSF or in PWH on ART versus controls in either compartment (χ^2^ test; *P* > 0.05).

### Longitudinal changes in blood and CSF TCR repertoire before and after ART.

T cell migration into the CNS from the periphery occurs under normal physiologic conditions, and clonally expanded T cells are found in the CSF of healthy individuals (29). However, it is not known how chronic HIV infection and how the initiation of ART and viral suppression affect T cell trafficking dynamics into the CSF. We tracked the longitudinal TCR repertoires of a PWH (P1) who underwent serial CSF and blood sampling, both before and at 3 time points after ART initiation (3, 7, and 9 months on ART). In this participant, we detected HIV RNA–producing cells in the CSF at the first 3 time points and in the blood at only the first (pre-ART) time point (Supplemental Table 5). Five of 28 T cell clones containing infected cells were found across longitudinal time points, and 3 of these clones were also detected between tissue compartments (Supplemental Figure 6).

**Figure 4:**
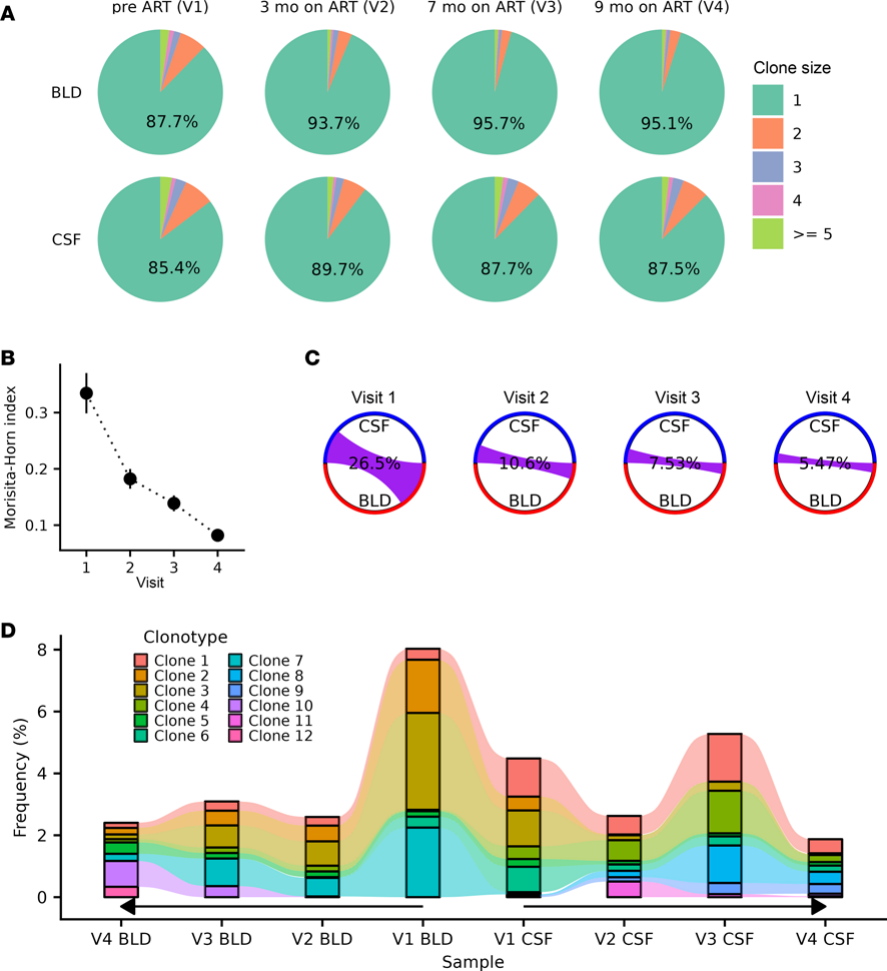
Longitudinal changes in TCR repertoire before and after ART. (**A**) TCR clone size proportion for participant P1 who was longitudinally sampled for 4 visits: pre-ART (V1) and 3, 7, and 9 months after ART (V2–V4). The frequency of the singleton clones is indicated. The number of T cells in each sample ranges from 1,564 to 15,315 for blood and between 2,212 and 3,780 for CSF. (**B**) MH overlap index between blood and CSF TCR clones of each visit. Higher values (closer to 1) indicate greater overlap. (**C**) Circos diagram showing the clonal overlap between blood and CSF T cells for P1 across time points. The purple chords correspond to cells in shared clones between blood and CSF. (**D**) Frequency of the largest 12 clones tracked across the visits and compartments. Note that time points were shown in reverse order for blood samples.

Next, to understand whether there are changes in distribution of T cell clones between tissues after the initiation of ART, we examined the clonality shifts in TCR repertoires across compartments and time points (Figure 4A). In both blood and CSF, we found a sharp decline in the frequency of expanded clones between pre-ART and 3 months on ART. For instance, before ART, nonsingleton clones (clones with multiple T cells) accounted for 12.3% and 14.6% of TCRs in the blood and CSF, respectively. However, 3 months after ART, these percentages declined to 6.3% and 10.3%, respectively. After ART, the clone size distribution remained consistent within blood and CSF compartments, with a small decrease in clonality across 3 months to 9 months after ART.

We next asked whether clonal overlap between blood and CSF compartments changed after ART. We evaluated the overlap of T cell clones between blood and CSF, finding a progressive decrease in MH overlap index from 0.33 to 0.08 across time points (Figure 4B) and a decrease in the percentage of cells in the clones shared between compartments from 26.5% to 5.47% (Figure 4C). This suggests the presence of more compartment-specific clones after ART compared with before ART.

To determine the dynamics of T cell clonal expansion after viral suppression, we next performed a longitudinal analysis of the TCR repertoire in CSF and blood before and after ART. We focused on the 12 T cell clones that were the most expanded in blood or CSF (Figure 4D). None of these 12 clones contained any infected cells. Prior to ART, these largest 12 clones constituted 8.0% and 4.5% of blood and CSF repertoire, respectively. Seven of the 12 large clones were shared between compartments (Supplemental Table 6). Following ART, contractions of the most expanded clones were observed for both the blood and CSF, though CSF T cell clones remained larger (less contracted) compared with those in the blood. Four clones remained expanded in the CSF, with limited or diminishing presence in blood after ART. Three months after ART initiation, 1 clone (Clone 11; TCRalpha [TRA]: CAASIGTTGANSKLTF, TCRbeta [TRB]: CASSPLQRHTDTQYF) emerged as a CSF-only clone; this clone was not found in the blood at any time point we tested. Lastly, we queried VDJdb to determine if any TRA or TRB sequences matched known specificities. We found that TRB CDR3 (CASSPPTGSNTEAFF) of a blood-unique clone (Clone 10) matches a TCR that targets the human BST2 gene, while a TRA CDR3 sequence (CAASGGGADGLTF) of a CSF-unique clone (Clone 8) has a match with multiple TCRs with targets for the human BST2 gene, Epstein-Barr virus (EBV) BZLF-1, and CMV IE1 (Supplemental Table 7).

## Discussion

The HIV reservoir persists during ART at diverse anatomical sites, including the CNS, posing a major barrier to HIV cure. Previous studies (28, 33) advanced our comprehension of the clonal expansion of the HIV reservoir in peripheral blood by examining clonality in infected T cell clones through single-cell transcriptome analysis of blood T cells. However, a critical gap in our knowledge remained, concerning the extent of clonal expansion of infected cells in the CNS during ART-treated chronic HIV infection. Here, we profiled single-cell transcriptomes and paired TCR repertoires of CSF and blood samples from PWH to gain preliminary insights into the dynamics of rare transcriptionally active HIV-1 RNA–producing T cells in both compartments and under the pressure of ART. By examining T cell clones across tissue compartments in PWH on ART, we found evidence for expanded T cell clones containing infected cells that were identical across tissue compartments, suggesting that the maintenance and expansion of infected T cell clones contributes to the CNS reservoir.

Importantly, by harnessing the increased resolution of using single-cell transcriptomes, traces of HIV-1 RNA were predominantly found in CD4 TCM in both compartments but more frequently in CSF than blood for 3 of 7 participants on ART who were on long-term suppressive ART. The gene expression of these infected cells in the CSF showed upregulation in key pathways, highlighting potential cellular mechanisms of HIV persistence and immune evasion. We extend prior studies in blood that have shown that single-cell technologies can identify transcriptionally active HIV-infected cells (33, 34).

When comparing the T cell clones between blood and CSF in PWH on ART, we found overlap of infected clones across the 2 compartments in a subset of participants, emphasizing that HIV-infected T cells are sometimes shared across anatomic sites but are, at other times, compartment specific. Our findings are consistent with prior findings, focused on viral sequences, that find identical HIV genomes in distant compartments and that find evidence for clonal expansion of infected cells within the CNS (6, 35). Our observation was made despite not enriching for specific memory CD4 T cells and profiling thousands of cells in the CSF and blood. It is important to note that most of the individuals we profiled did not have shared infected clones across the blood and CSF compartments; thus, further study is required to understand the clinical factors that may contribute to the dynamics of T cell trafficking and clonal expansion in different PWH.

Focusing on 1 participant who was sampled longitudinally, we noted that ART initiation shifted the T cell clonal landscape both within and between the blood and CSF. ART is associated with contraction of all expanded clones in the blood and the CSF, though the degree of contraction was less robust in the CSF compared with blood, suggesting ongoing antigen stimulation in the CNS compartment. We also noted the emergence of an expanded T cell clone in the CSF after several months of ART. While we could not determine the antigen specificity of this TCR, the presence of a robust clonal expansion in the CSF again suggests continual antigen stimulation in the CNS, possibly by HIV and despite ART.

While our study provides valuable insights, there are inherent limitations. One surprising observation from the study was the detection of HIV reads in several non-CD4 immune cells, including 9 CD8 T cells, 3 B cells, and 1 DC. This presence of viral RNA may be attributed to the inherent phagocytic capabilities of B and DCs. However, technical intricacies, such as potential issues with multiplets or errors in cell type identification, cannot be excluded (36). In addition, the 10× sequencing platform employed does not yield full-length viral sequences; thus, we are unable to ascertain HIV-1 splicing dynamics and replication competence of the detected infected cells. Furthermore, we noticed that utilizing the autologous sequence enabled detection of 2% more HIV reads (3 more infected cells) in participant P1 compared with reads when employing reference sequences, suggesting the potential that some HIV-infected cells are not identified using the standard, reference genome based approaches. However, since the plasma and CSF HIV-1 RNA levels in other PWH were undetectable, we could not source the autologous sequence. In addition, for these PWH that were on suppressive ART, our ability to characterize the gene expression and compartmentalization of the infected cells is limited because of the rarity of these cells. It is also important to note that participant P1 was treated for neurosyphilis after the initial visit; thus, shifts in the TCR repertoire of this individual were likely also affected by treatment of neurosyphilis. Another limitation is the lack of precise information concerning the antigens targeted by persistent TCR clones. Publicly available TCR-antigen databases could only annotate, on average, 4.1% and 11.2% of the TCRβ and TCRα sequences, respectively. The future of TCR studies holds promise with the development of computational tools that infer antigen specificity to the TCR; this would substantially enhance our understanding. Finally, our study focused on CSF, a CNS component that is readily accessible in living PWH. However, CSF cells do not represent the entire intricacy of the viral reservoir in the brain.

## Methods

### Sex as a biological variable.

Our study examined both male and female participants recruited from the regions surrounding New Haven, Connecticut, USA, and consented to the HIV Associated Reservoirs and Comorbidities (HARC) Cohort Study at Yale (HIC #1502015318)). Demographic and clinical information regarding all participants is listed in Supplemental Table 1.

### Patient selection and sample collection.

Asymptomatic PWH (P2–P8) were enrolled (*n* = 7), and all were on stable ART (median 16 years) with suppressed plasma HIV-1 RNA levels (<50 copies/mL) and a median CD4 T cell count of 503 cells/μL. One additional PWH was enrolled (P1) with newly diagnosed HIV and AIDS, not yet on ART, with a CD4 T cell count of 47 cells/μL and a plasma HIV-1 RNA level of 257,000 copies/mL. Control participants were healthy volunteers recruited from the surrounding community for research sampling (*n* = 6) plus 1 hospitalized individual C1 undergoing a workup for gait instability. All control participants were confirmed HIV^–^ by plasma enzyme immunoassay and were screened for confounding neurological conditions. All participants consented to lumbar puncture (up to 30 cc CSF removed) and blood draw for research purposes, or they consented to donate additional CSF and blood samples collected during clinical standard-of-care procedures. Participant P1 underwent longitudinal study visits and consented to provide blood and CSF samples at HIV diagnosis/baseline and 3, 6, and 9 months after initiating ART.

### Emulsion-based single-cell library preparation.

Fresh CSF and blood were processed within 1 hour of collection. CSF was centrifuged at 300*g* for 10 minutes at room temperature (25°C). The supernatant was aspirated to 39 μL, and the pelleted cells were used for scRNA-Seq. Peripheral blood mononuclear cells (PBMCs) were isolated from whole blood via Ficoll gradient with SepMate tubes (Stemcell Technologies) and were resuspended in 0.04% PBS-BSA for scRNA-Seq. For blood, approximately 8,000 single cells per sample were processed using the Chromium 5′ Single-Cell Gene Expression system (10× Genomics). For CSF, the entire cell pellet was processed for 10×. Samples were sequenced on Illumina HiSeq 4000 or Illumina HiSeq2500 at an average depth of 30,000–50,000 reads per cell.

### Processing of 10× Genomics single-cell 5′ gene expression data.

Base calls were converted to FASTQ sequences and demultiplexed using the Cell Ranger 7.0.0 (37) mkfastq function. Demultiplexed FASTQ reads were aligned to the coding sequences of the GCRhg38 coding genome supplied by 10× Genomics. The sequence depth information for GEX, BCR, and TCR data is available in Supplemental Tables 3 and 4. We used the Cell Ranger count subcommand to generate the count matrices, barcode assignments, and feature calls. Seurat v4 (38) was used for gene expression analysis. We removed cells with fewer than 400 transcripts or with mitochondrial content of more than 15% of all transcripts. We library-normalized and log-transformed the UMI counts and selected the top 2,000 variable genes using the “FindVariableFeatures” function with the “vst” option. We removed immunoglobulin and TCR-related genes from the list of highly variable genes so that their properties could be analyzed independently of cell type annotation. We centered and scaled the data and ran the principal component analysis (PCA) with 50 PCs to generate the Uniform Manifold Approximation and Projection (UMAP) embeddings. We annotated the cell type using Azimuth v0.4.5 (31) with human PBMC reference v1.0.0 (celltype.l2 level annotation). In addition to the cell type provided by the PBMC reference, we annotated CD204^+^ CSF microglia-like cells using ScType with markers (MSR1, APOE, CTSB, APOC1, AXL, and TREM2) as reported in ref. 39.

### Processing 10× genomics single-cell TCR data.

We used Cell Ranger 7.0.0 vdj subcommand to assemble TCR sequences and call clonotypes, followed by aggr subcommand to perform clonotype grouping across samples for each donor. To quantify the clonal overlap between samples, we randomly downsampled each sample to the minimum number of cells across samples and computed the MH overlap index between pairs of blood and CSF samples for each donor using R package abdiv 0.2.0. We visualized clonal sharing between blood and CSF using circus plot produced by R package circlize 0.4.5 and alluvial plot from scRepertoire 1.10.1.

### Identifying HIV-1 RNA^+^ cells.

As has been previously reported (33, 40), we aligned scRNA-Seq reads for P2–P8 against a clade B HIV-1 consensus reference sequence (GenBank: K03455.1) by using a custom reference generated from Cell Ranger 7.0.0 mkref concatenating the HIV-1 sequence to the prebuilt GRCh38 human reference genome. We categorized cells with at least 3 HIV-1 reads as HIV-RNA^+^ and detected no HIV-1 reads in all control samples. Additionally, we obtained an autologous HIV-1 sequence for P1 from pre-ART plasma using near–full-length sequencing. To obtain the autologous HIV-1 sequence for P1, the viral particles were pelleted at 24,000 g/1 hour, viral RNA was extracted, and cDNA was generated and amplified in 2 fragments (5′ half and 3′ half of HIV-1 genome) using a 1-step reverse transcription PCR protocol. Amplicons were sequenced by Illumina platform and a near–full-length contig generated using Sequencher v5.4.6. This autologous HIV-1 reference was utilized to identify HIV-1 RNA^+^ cells in P1. An increase of 3 (8.8%) infected cells was obtained by utilizing a consensus sequence for P1 versus a consensus HIV-1 reference sequence K03455.1.

### Statistics.

In our study, we employed the χ^2^ test to compare cell type frequency differences as well as epitope species composition between blood and CSF. We used Wilcoxon ranked-sum tests to compare the frequency differences between compartments for individual cell type, with Bonferroni correction for multiple testing. We also used Wilcoxon ranked-sum tests with Bonferroni correction to find differentially expressed genes between infected and uninfected CD4 TCM. Additionally, we utilized the Welch 2-sample, 2-tailed *t* test to assess differences in the MH index between PWH on ART and HIV-uninfected controls.

### Data availability.

The processed data were deposited on GEO under accession no. GSE243905 for C4–C6 and P1–P8. The data for control C1–C3 were previously published on SRA (study: PRJNA717310, C1_BLD_RNA: SRR14076861; C1_BLD_TCR: SRR14076833; C1_CSF_RNA: SRR14076871; C1_CSF_TCR: SRR14076842; C2_BLD_RNA: SRR14076860; C2_BLD_TCR: SRR14076832; C2_CSF_RNA: SRR14076869; C2_CSF_TCR: SRR14076841; C3_BLD_RNA: SRR14076858; C3_BLD_TCR: SRR14076831; C3_CSF_RNA: SRR14076868; and C3_CSF_TCR: SRR14076840). The scripts for the analysis and the figures are available at https://bitbucket.org/mamiewang/farhadian_hiv/ Values for all data points in graphs are reported in the Supporting Data Values file.

### Study approval.

Written informed consent was obtained from all participants under approved human research ethics committee protocols from the Yale IRB (HIC 1502015318).

## Author contributions

SFF, SS, JY, HR, LAB, MF, and JC acquired samples. MW, JY, HR, BD, JC, JM, EKH, JC, SFF conducted experiments and acquired data. MW, YK, SS, MJC, and SFF analyzed and interpreted data. MW, MJC, and SFF wrote the manuscript with input from all authors. SFF designed and oversaw the research study.

## Supplementary Material

Supplemental data

Supporting data values

## Version 1 04/08/2024

Electronic publication
