# Supplementary material for: HIV-1–infected T cell clones are shared across cerebrospinal fluid and blood during ART
Source: JCI Insight. 2024 Apr 8;9(7):e176208. doi: 10.1172/jci.insight.176208 (PMC11128194; doi:10.1172/jci.insight.176208)
Supplement: Supplemental data [file jciinsight-9-176208-s080.pdf]

Supplemental information

ID	Sex	Age	Ethnicity	Race	Cohort	ART regimen
P1	Male	38	Not Hispanic or Latino	Asian	Chronic HIV, newly diagnosed with comorbid neurosyphilis	None; then ABC/3TC/DTG
P2	Male	47	Not Hispanic or Latino	White	Chronic HIV on ART	TAF/FTC/BIC
P3	Male	37	Not Hispanic or Latino	Black or African American	Chronic HIV on ART	EVG/COBI/FTC/TAF
P4	Male	56	Not Hispanic or Latino	Black or African American	Chronic HIV on ART	DTG/3TC
P5	Male	62	Not Hispanic or Latino	Black or African American	Chronic HIV on ART	ABC/3TC/DTG
P6	Female	61	Not Hispanic or Latino	Black or African American	Chronic HIV on ART	DTG/RPV
P7	Male	59	Not Hispanic or Latino	White	Chronic HIV on ART	TAF/FTC/BIC
P8	Male	34	Not Hispanic or Latino	White	Chronic HIV on ART	DRV/COBI, TAF/FTC/RPV, DTG
C1	Male	75	Not Hispanic or Latino	White	HIV-uninfected	n/a
C2	Male	54	Not Hispanic or Latino	Black or African American	HIV-uninfected	n/a
C3	Male	54	Not Hispanic or Latino	Black or African American	HIV-uninfected	n/a
C4	Female	48	Not Hispanic or Latino	Black or African American	HIV-uninfected	n/a
C5	Male	31	Hispanic or Latino	White	HIV-uninfected	n/a
C6	Male	62	Not Hispanic or Latino	Black or African American	HIV-uninfected	n/a

Table S1. Subject demographics.

Subject	Visit ID	Time on ART	Plasma HIV-1 RNA (copies/mL)	CSF HIV-1 RNA (copies/mL)	CD4 Count
P1	V1	0	257,000	49,800	46
P1	V2	3 months	<20	762	104
P1	V3	7 months	<20	<20	248
P1	V4	9 months	21.6	<20	158
P2	V3	11 years	<20	Not Detected	929
P3	V5	16 years	<20	Not Detected	495
P4	V8	12 years	Not Detected	Not Detected	359
P5	V7	26 years	Not Detected	Not Detected	503
P6	V1	21 years	Not Detected	Not Detected	740
P7	V3	24 years	Not Detected	Not Detected	511
P8	V1	14 years	45.1	<20	491

Table S2. Plasma HIV-1 RNA and CD4 T cell count of People with HIV.

Sample	Estimated Number of Cells	Mean Reads per Cell	Median Genes per Cell	Fraction Reads in Cells
C1_1_1_BLD_RNA	7,843	52,681	1,573	92.10%
C1_1_1_CSF_RNA	7,398	51,011	1,796	95.30%
C2_1_6_BLD_RNA	6,270	64,775	1,404	89.10%
C2_1_6_CSF_RNA	4,275	101,700	1,577	86.20%
C3_1_2_BLD_RNA	9,837	41,142	1,465	92.20%
C3_1_2_CSF_RNA	3,793	88,408	1,442	73.80%
C4_1_1_BLD_RNA	7,887	45,759	1,627	93.80%
C4_1_1_CSF_RNA	4,554	101,567	1,678	87.80%
C4_2_1_BLD_RNA	8,734	44,086	1,676	94.00%
C5_1_2_BLD_RNA	8,339	46,902	1,892	94.50%
C5_1_2_CSF_RNA	7,241	63,694	1,947	94.80%
C5_2_2_BLD_RNA	12,505	23,636	1,604	96.70%
C6_1_1_BLD_RNA	572	609,092	2,450	94.30%
C6_1_1_CSF_RNA	5,591	51,743	1,567	92.20%
C6_2_1_BLD_RNA	5,860	58,490	1,840	95.30%
P1_1_1_BLD_RNA	5,843	40,527	2,057	94.40%
P1_1_1_CSF_RNA	7,853	26,682	1,678	95.80%
P1_1_2_BLD_RNA	7,187	38,630	1,799	93.10%
P1_1_2_CSF_RNA	5,300	51,155	1,687	95.90%
P1_1_3_BLD_RNA	4,441	23,955	1,668	93.80%
P1_1_3_CSF_RNA	4,168	23,964	1,486	90.90%
P1_1_4_BLD_RNA	37,781	8,201	859	89.30%
P1_1_4_CSF_RNA	4,174	69,341	1,956	92.10%
P2_1_3_BLD_RNA	9,832	24,011	1,479	95.50%
P2_1_3_CSF_RNA	7,191	31,969	1,441	96.00%
P3_1_5_BLD_RNA	12,996	22,996	1,592	90.40%
P3_1_5_BLD_RNA_enriched	25,812	10,803	1,093	98.20%
P3_1_5_CSF_RNA	17,746	16,943	1,337	98.10%
P3_2_5_BLD_RNA	12,973	21,483	1,490	95.80%
P4_1_8_BLD_RNA	13,272	22,381	1,724	96.40%
P4_1_8_BLD_RNA_enriched	20,357	34,858	1,658	98.10%
P4_1_8_CSF_RNA	6,771	81,774	1,640	95.90%
P4_2_8_BLD_RNA	12,963	21,805	1,623	96.00%
P5_1_7_BLD_RNA	12,613	24,170	1,684	95.90%
P5_1_7_BLD_RNA_enriched	4,159	73,203	1,600	95.50%
P5_1_7_CSF_RNA	4,666	55,499	1,279	93.00%
P5_2_7_BLD_RNA	11,040	26,408	1,729	96.90%
P6_1_1_BLD_RNA	11,636	26,364	1,820	96.10%
P6_1_1_BLD_RNA_enriched	8,660	21,786	1,391	98.80%
P6_1_1_CSF_RNA	14,904	16,988	1,291	97.00%
P6_2_1_BLD_RNA	11,195	28,549	1,860	96.40%
P7_1_3_BLD_RNA	8,755	38,982	2,077	95.90%
P7_1_3_BLD_RNA_enriched	4,646	44,116	1,881	94.70%
P7_1_3_CSF_RNA	12,193	14,750	1,304	95.20%
P7_2_3_BLD_RNA	7,548	40,897	2,014	95.50%
P8_1_1_BLD_RNA	14,896	14,911	1,382	97.20%
P8_1_1_CSF_RNA	11,726	17,540	1,384	98.10%

Table S3. Summary statistics on the quality of single-cell RNA sequencing. Sample names follow the naming convention of subject_replicate_visit_tissue_RNA. Any sample with suffix “enriched” means that it has been enriched for CD4+ cells.

Sample	Estimated Number of Cells	Mean Read Pairs per Cell	Number of Cells with Productive V J Spanning Pair
C1_1_1_BLD_TCR	3,053	5,736	2,508
C1_1_1_CSF_TCR	4,780	3,291	4,219
C2_1_6_BLD_TCR	2,288	8,602	1,515
C2_1_6_CSF_TCR	3,130	4,550	2,810
C3_1_2_BLD_TCR	5,729	3,095	4,072
C3_1_2_CSF_TCR	3,147	4,832	2,521
C4_1_1_BLD_TCR	4,657	22,252	4,201
C4_1_1_CSF_TCR	3,775	23,636	3,331
C4_2_1_BLD_TCR	5,112	18,971	4,634
C5_1_2_BLD_TCR	4,484	18,197	3,907
C5_1_2_CSF_TCR	3,905	21,675	3,407
C5_2_2_BLD_TCR	11,363	6,096	9,789
C6_1_1_BLD_TCR	399	258,968	287
C6_1_1_CSF_TCR	4,455	15,016	3,787
C6_2_1_BLD_TCR	2,837	30,564	2,505
P1_1_1_BLD_TCR	2,267	24,106	2,109
P1_1_1_CSF_TCR	5,599	9,612	5,085
P1_1_2_BLD_TCR	4,322	18,954	3,991
P1_1_2_CSF_TCR	4,342	17,437	3,906
P1_1_3_BLD_TCR	1,681	21,638	1,428
P1_1_3_CSF_TCR	3,052	13,893	2,550
P1_1_4_BLD_TCR	18,185	4,809	13,684
P1_1_4_CSF_TCR	3,524	21,048	3,172
P2_1_3_BLD_TCR	5,574	14,710	4,748
P2_1_3_CSF_TCR	6,535	12,384	5,741
P3_1_5_BLD_TCR	6,449	16,496	5,492
P3_1_5_BLD_TCR_enriched	22,550	3,418	17,784
P3_1_5_CSF_TCR	15,130	4,463	12,150
P3_2_5_BLD_TCR	6,985	14,903	5,900
P4_1_8_BLD_TCR	7,160	14,378	6,357
P4_1_8_BLD_TCR_enriched	17,468	7,853	14,984
P4_1_8_CSF_TCR	6,099	25,499	5,022
P4_2_8_BLD_TCR	6,494	19,676	5,769
P5_1_7_BLD_TCR	5,534	16,259	4,868
P5_1_7_BLD_TCR_enriched	3,871	20,602	3,353
P5_1_7_CSF_TCR	4,095	21,163	3,516
P5_2_7_BLD_TCR	4,832	22,310	4,374
P6_1_1_BLD_TCR	6,886	14,758	6,021
P6_1_1_BLD_TCR_enriched	8,213	9,841	7,352
P6_1_1_CSF_TCR	13,313	6,096	11,748
P6_2_1_BLD_TCR	6,570	14,852	5,870
P7_1_3_BLD_TCR	4,563	20,372	4,155
P7_1_3_BLD_TCR_enriched	4,560	5,922	4,131
P7_1_3_CSF_TCR	10,431	4,637	8,877
P7_2_3_BLD_TCR	3,775	24,392	3,430
P8_1_1_BLD_TCR	9,442	4,021	8,040
P8_1_1_CSF_TCR	10,637	4,285	9,298

Table S4. Summary statistics on the quality of single-cell TCR sequencing. Sample names follow the naming convention of subject_replicate_visit_tissue_TCR. Any samples with suffix “enriched” means that it has been enriched for CD4+ cells.

Sample	BLD	CSF
P1_v1	2	32
P1_v2	0	1
P1_v3	0	2
P1_v4	0	0
P2	0	0
P3	12	10
P4	10	3
P5	1	2
P6	0	0
P7	1	1
P8	2	6
Total	28	57

Table S5. The number of cells with HIV transcripts detected per sample.

TRA	TRB	V1_BLD	V1_CSF	V2_BLD	V2_CSF	V3_BLD	V3_CSF	V4_BLD	V4_CSF
CAASERDSNYQLIW	CASSVAWASTDTQYF	0.35%	1.23%	0.28%	0.60%	0.30%	1.54%	0.16%	0.45%
CAFIIQGAQKLVF	CASSLLAGGPKSYEQYF	1.72%	0.45%	0.51%	0.02%	0.48%	0.00%	0.21%	0.03%
CAGEPAYSGTYKYIF	CASDVPGQGVEKLFF	3.13%	1.16%	0.79%	0.16%	0.71%	0.29%	0.15%	0.03%
CALNNQGGKLIF	CASSEDRTNQPQHF	0.04%	0.41%	0.19%	0.67%	0.18%	1.38%	0.10%	0.23α%
CAYRRLGNTPLVF	CASSFLDGQGKDTDTQYF	0.18%	0.25%	0.19%	0.12%	0.18%	0.10%	0.37%	0.11%
CIVRPVGGTSYGKLTF	CASSLTSTDTQYF	0.35%	0.82%	0.02%	0.21%	0.00%	0.29%	0.00%	0.20%
CLLGPPFGNEKLTF	CASSQDAGPGQTTDTQYF	2.25%	0.07%	0.60%	0.00%	0.89%	0.00%	0.23%	0.00%
CAASGGGADGLTF	CSVFIGTSTYEQYF	0.00%	0.04%	0.00%	0.21%	0.00%	1.21%	0.00%	0.40%
CAGARGNKLVF	CSARDGGLAGEQFF	0.00%	0.05%	0.00%	0.14%	0.00%	0.36%	0.00%	0.31%
CAVNERRLTGGGNKLTF	CASSPPTGSNTEAFF	0.00%	0.00%	0.02%	0.00%	0.36%	0.00%	0.84%	0.09%
CAASIGTTGANSKLTF	CASSPLQRHTDTQYF	0.00%	0.00%	0.00%	0.51%	0.00%	0.10%	0.00%	0.03%
CAASEVGLTF	CSARDFGRSSGRGEQYF	0.00%	0.00%	0.00%	0.00%	0.00%	0.00%	0.34%	0.00%

Table S6. The frequency of the top twelve largest clones across samples. TRA: TCRα CDR3 sequence, TRB: TCRβ CDR3 sequence. V[n]_[BLD|CSF]: Visit n blood or CSF sample.

Gene	CDR3	V	J	MHC A	MHC class	Epitope	Epitope gene	Epitope species
TRB	CASSPPTGSNTEAFF	TRBV6-6*01	TRBJ1-1*01	HLA-A*02	MHCI	LLLGIGILV	BST2	Homo Sapiens
TRA	CAASGGGADGLTF	TRAV29/DV5*01	TRAJ45*01	HLA-A*02	MHCI	LLLGIGILV	BST2	Homo Sapiens
TRA	CAASGGGADGLTF	TRAV29/DV5*01	TRAJ45*01	HLA-A*02	MHCI	LLLGIGILV	BST2	Homo Sapiens
TRA	CAASGGGADGLTF	TRAV29/DV5*01	TRAJ45*01	HLA-B*08:01	MHCI	RAKFKQLL	BZLF1	EBV
TRA	CAASGGGADGLTF	TRAV29/DV5*01	TRAJ45*01	HLA-A*03:01	MHCI	KLGGALQAK	IE1	CMV

Table S7. VDJdb CDR3 exact matches with two of the twelve top clones across samples.

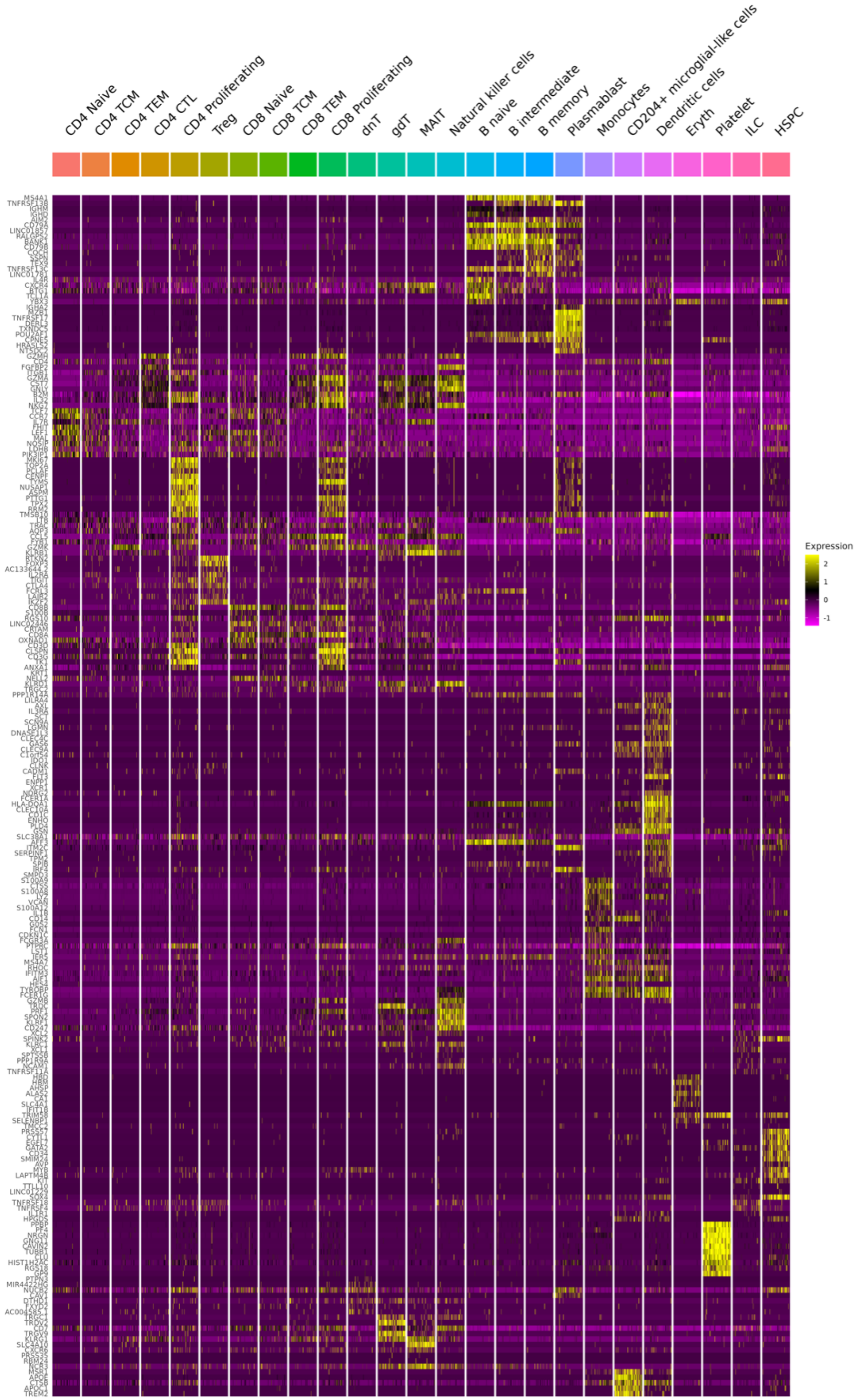

Figure S1. Cell type markers for annotation.

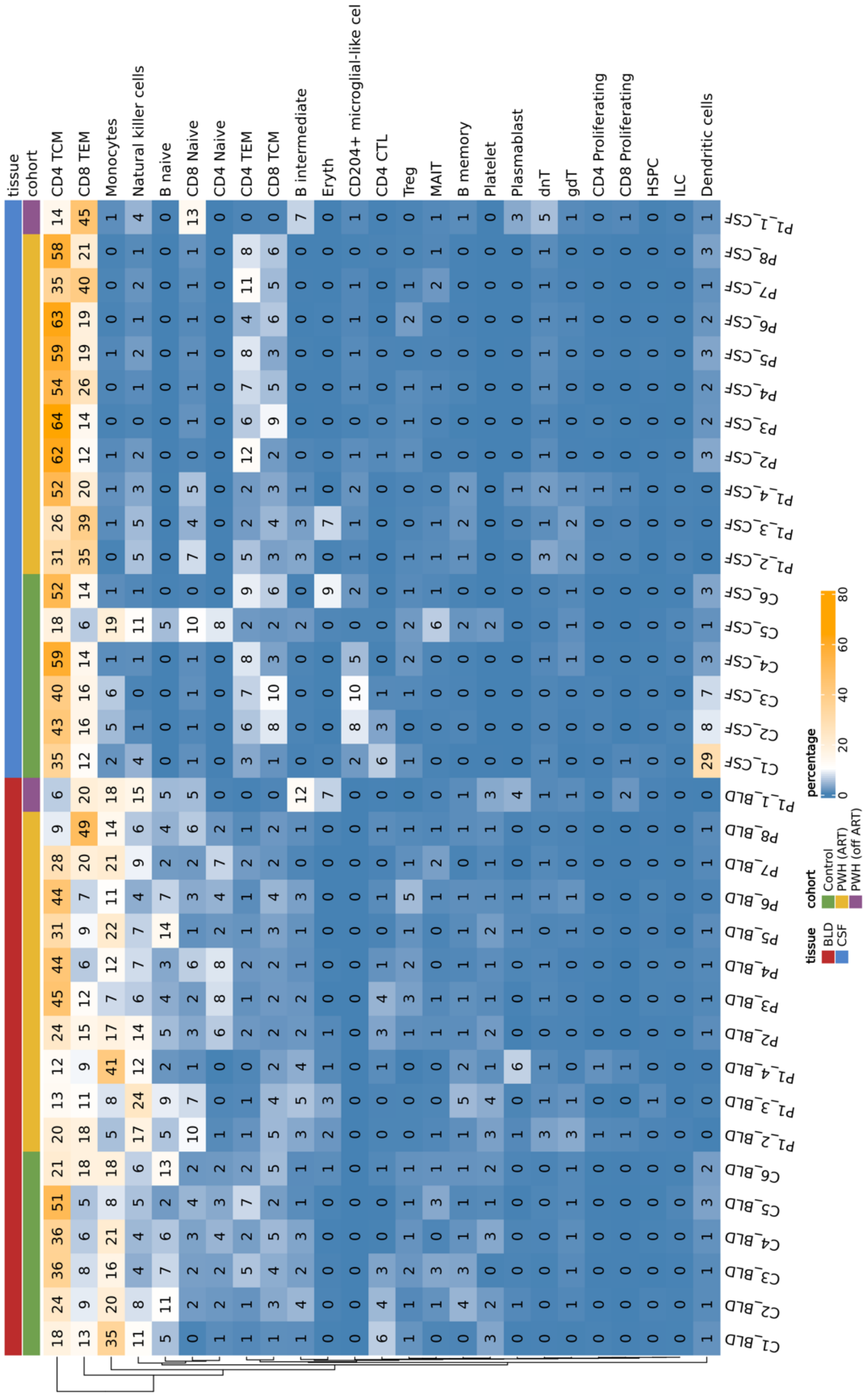

Figure S2. Cell type frequency composition. Each column listed the percentage of each cell type within the single-cell RNA sequencing samples, with the top two bars annotating the tissue and cohort where the sample comes from.

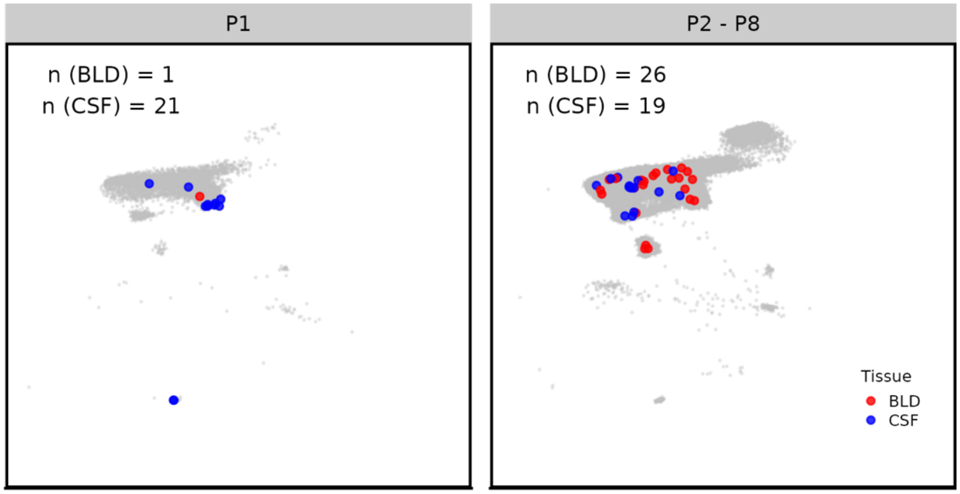

Figure S3. UMAP of CD4 T cells with HIV transcripts detected (left: PWH P1, right: PWH P2 – P8). The colored dots indicate CD4 T cells with HIV transcripts detected (blue: CSF, red: peripheral blood). The total numbers of infected cells in each tissue compartments are shown on the top left corner of each panel.

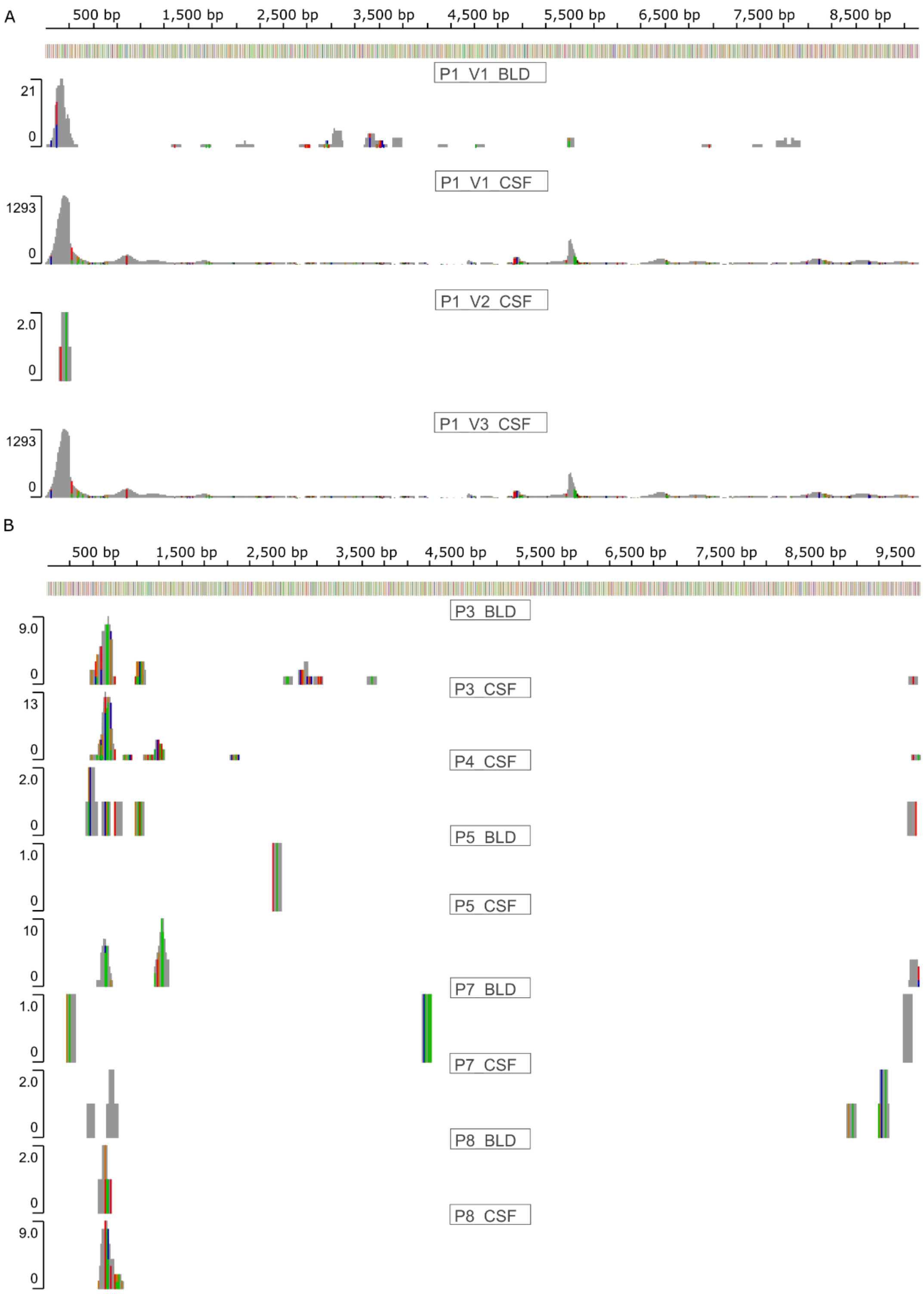

Figure S4. Coverage of aligned HIV reads (A) P1 HIV autologous sequence (B) HXB2 HIV-1 sequence (GenBank. K03455.1)

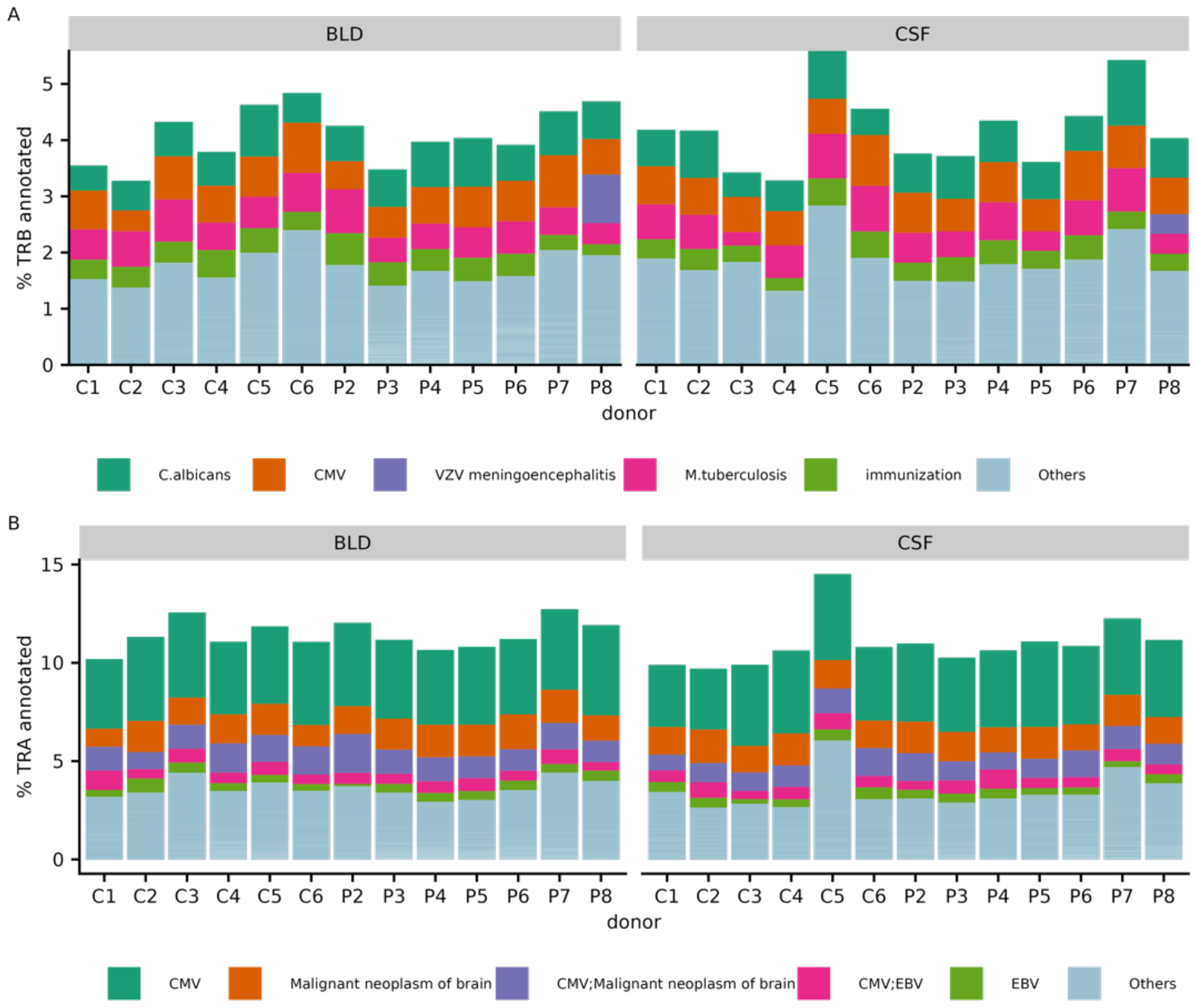

Figure S5. Top epitope species annotated by exact match of TCRB (A) and TCRA (B) CDR3 from public TCR specificity databases.

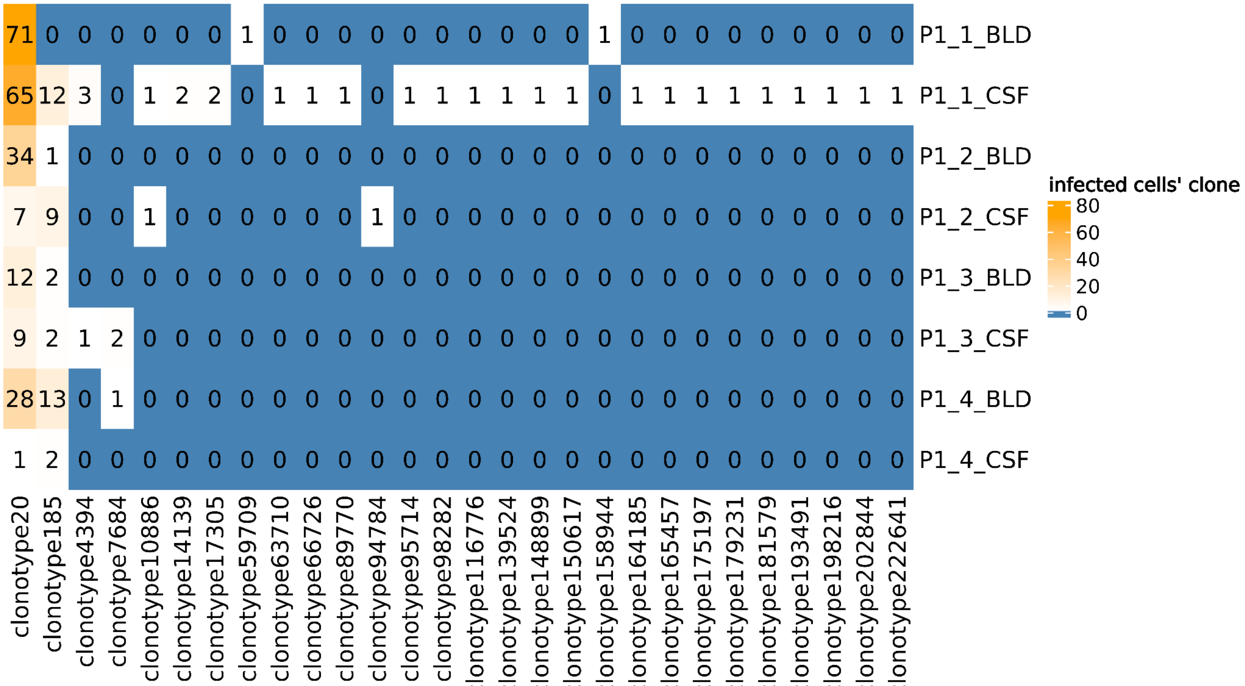

Figure S6. Total number of cells in infected T cell clones for P1. The number and color of the heatmap indicates the number of cells in the T cell clones that contain at least one cell with HIV transcripts detected in each sample. Each column corresponds to a T cell clone and each row is a sample.
